# Osteochondral Avulsion Fracture of the Femoral Origin of the Anterior Cruciate Ligament in an 11-Year-Old Child

**DOI:** 10.1155/2012/506798

**Published:** 2012-05-20

**Authors:** Sunil Kumar Pai, Nayef Aslam Pervez, Graham Radcliffe

**Affiliations:** The Department of Orthopaedics, Bradford Royal Infirmary, Duckworth Lane, Bradford, West Yorkshire BD9 6RJ, UK

## Abstract

Although tibial end avulsions of the anterior cruciate ligament are relatively common in clinical practice, avulsions of the femoral end of this ligament are by comparison rare. We present the case of an 11-year-old boy with a bony avulsion injury, which was presumed to have arisen from the tibial insertion of the anterior cruciate ligament but turned out instead to be an osteochondral avulsion fracture of the femoral origin. This unexpected finding that was not detected during preoperative workup resulted in the first attempt at surgical fixation being aborted. The need for a second planned definitive fixation procedure emphasises the importance of combining a thorough history and examination in association with appropriate imaging in the patient workup. The patient's definitive operative treatment and outcome are described. Although rare, surgeons (and emergency room doctors) treating such patients should include femoral end avulsion injuries of the anterior cruciate ligament in the differential diagnosis of a child presenting with an acute haemarthrosis of the knee. Furthermore, once diagnosed, early onward referral to an experienced knee surgeon is advocated.

## 1. Introduction

In the skeletally immature because ligamentous structures are stronger than their osseous attachments, intrasubstance ligament injuries are rare [1]; therefore, in the context of the paediatric knee, injuries that stress the anterior cruciate ligament (ACL) usually result in chondral or osteochondral avulsion fracture, characteristically from the tibial attachment of the ligament [2], resulting in a tibial spine avulsion fracture. By comparison, avulsions of the ACL from the femoral origin are rare, and to date only nine other cases of femoral end avulsion injuries of the ACL have been reported in the English-speaking literature. We present the case of an 11-year-old boy with a femoral ended osteochondral avulsion injury of the ACL. Despite their rare occurrence, femoral end avulsions of the ACL should be included in the differential diagnosis of a child with an acute haemarthrosis of the knee.

## 2. Case Presentation

**Figure 1:**
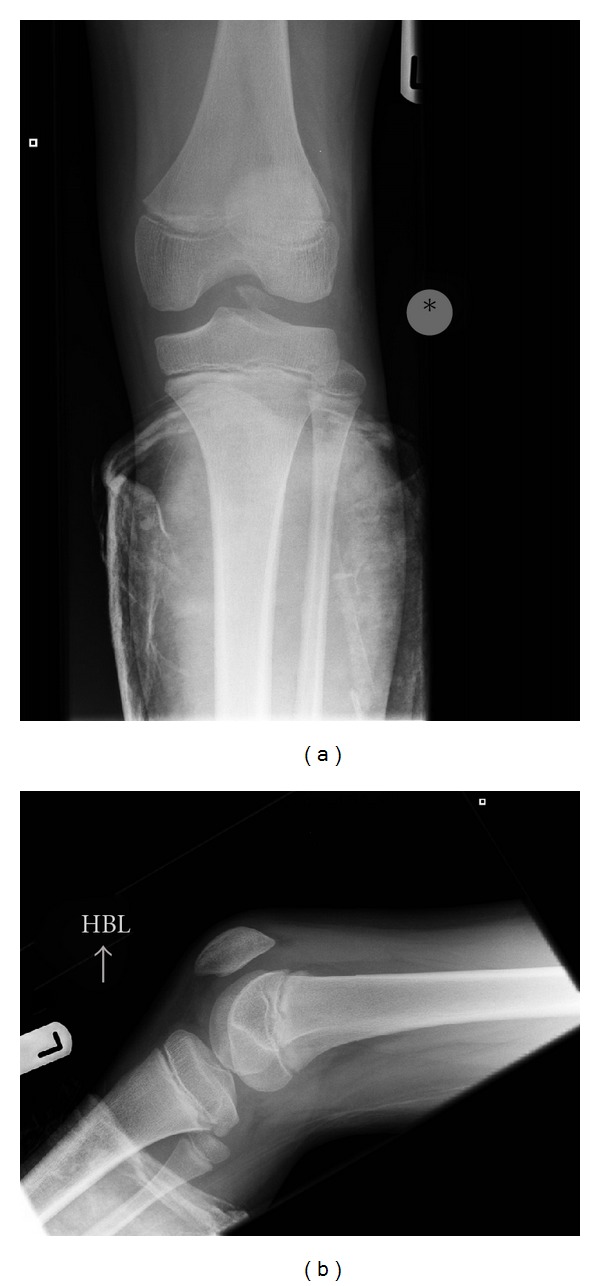
(a) Anteroposterior and (b) lateral views of the patient's knee depicting an intra-articular fracture fragment.

**Figure 2:**
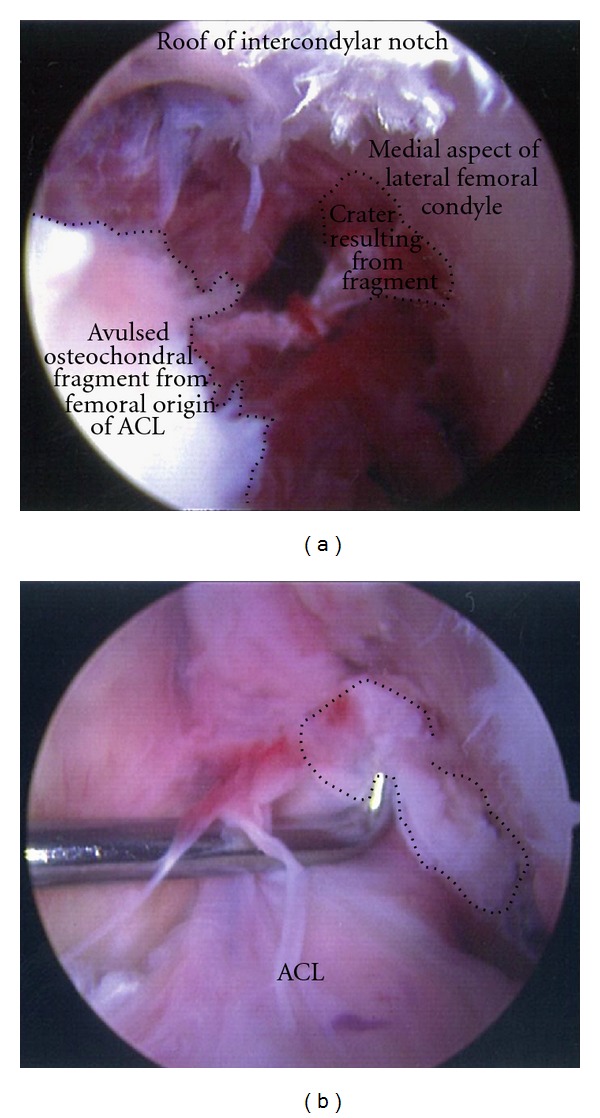
Intraoperative arthroscopic images depicting (a) an empty lateral wall of the intercondylar notch resulting from an osteochondral avulsion fracture of the femoral end of the ACL and (b) an arthroscopic hook reducing the fragment (outlined by black dots) to its normal position.

**Figure 3:**
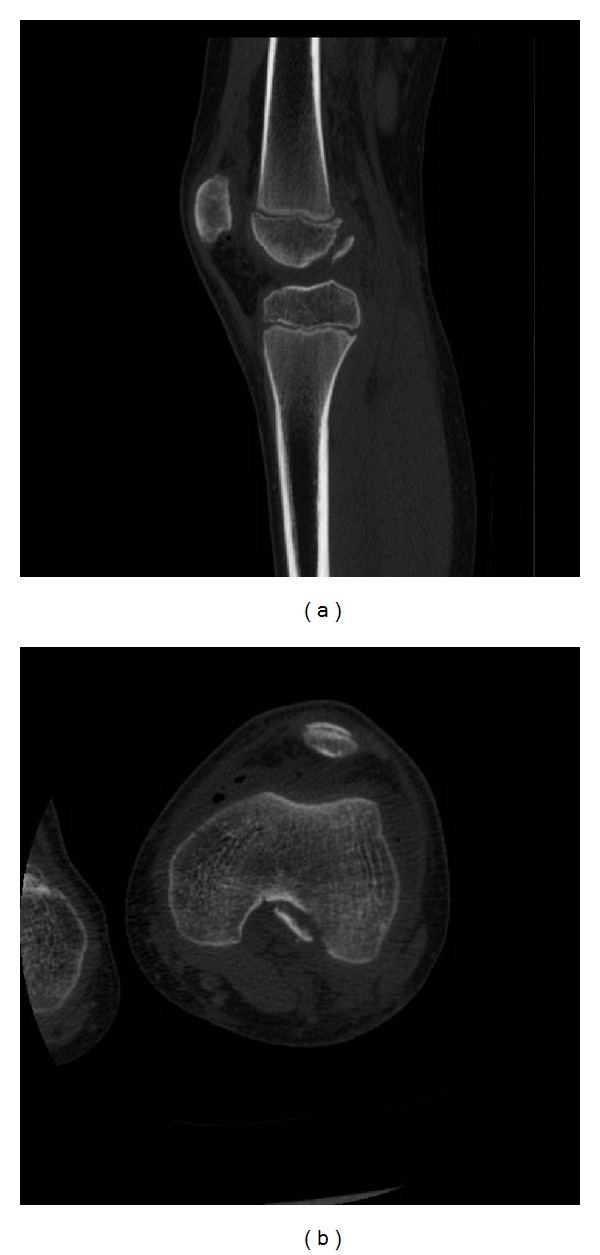
CT scan depicting (a) sagittal and (b) axial views of the patients knee depicting the avulsed osteochondral fragment.

**Figure 4:**
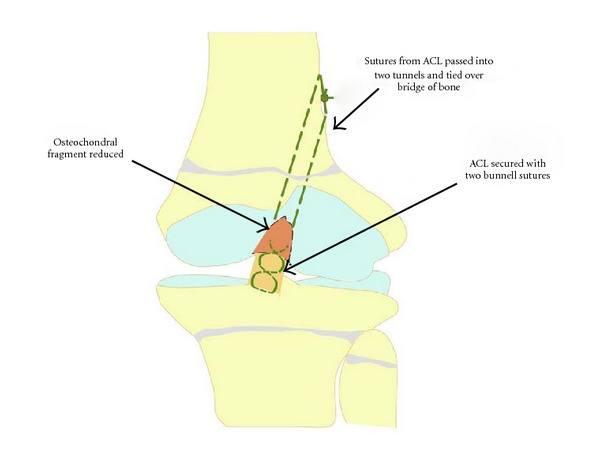
Schematic diagram depicting the method of repair.

**Figure 5:**
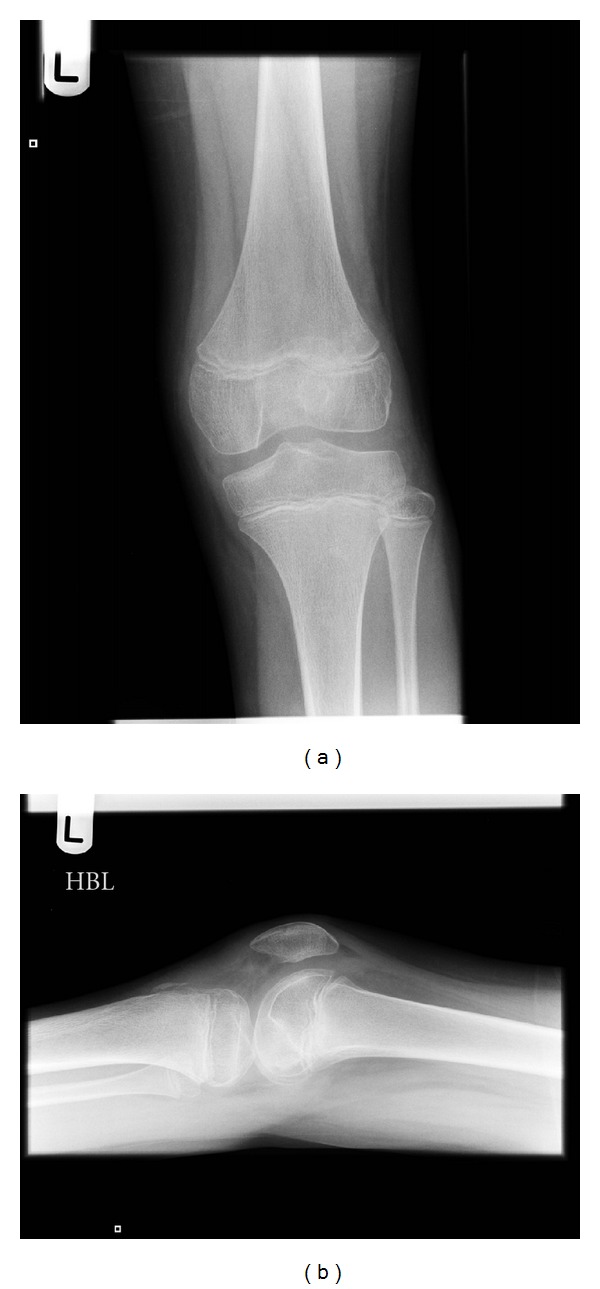
(a) Anteroposterior and (b) lateral views of the patients knee at three-month follow up.

An 11-year-old male patient presented to our institution after sustaining trauma to his left lower extremity. On the day of presentation, there had been high winds in the vicinity resulting in the corrugated iron roof of a garage he had been walking nearby blowing off and landing on his left leg. The patient was trapped under the sheet of metal until freed by attending paramedics. In the emergency department, primary and secondary survey assessments of the patient revealed injuries to the knee and distal fibula only. A full formal examination of the knee was not possible due to pain and a tense haemarthrosis. Subsequent plain radiographs revealed an intra-articular avulsion fracture (Figure 1), as well as fracture of the distal fibular diaphysis. A working diagnosis of displaced tibial spine avulsion fracture was made, and the patient was scheduled for surgery the following day to assess and fix the injury. Examination under anaesthesia revealed a ten-degree block to neutral extension of the knee and a positive Lachmann test with a soft end point. Arthroscopic examination of the knee revealed a lesion at odds with the preoperative diagnosis of tibial spine avulsion. A femoral end osteo-chondral avulsion fracture of the ACL was revealed (Figure 2). Due to the unexpected findings and uncertainty about the optimal method of fixation in a skeletally immature patient, the fragment was reduced back into its normal position, the arthroscopic portals were closed, and the knee was splinted in extension in a plaster of Paris splint. With the benefit of hindsight, the treating team acknowledged that if a bone fragment this large had been avulsed from the tibial insertion of the ACL, this should have been reflected in the architecture of the tibial spine footprint on the preoperative X-ray. But as the appearances of this region appeared normal, this avulsion was unlikely to have occurred from the tibial end of the ACL, rather occurring from the less common femoral origin. A CT scan was subsequently obtained (Figure 3), and the literature was scrutinised to aid preoperative planning of definitive fixation. Subsequent definitive fixation was perfomed nine days following the index procedure using a part arthroscopic and part open approach. Initial arthroscopic manoeuvres (including the use of a direct anterior portal in addition to the two standard portals) involved gentle curettage of the residual crater over the medial aspect of the lateral femoral condyle from where the ACL had been avulsed. Then two 2.4 mm Beath pins were passed in transosseous manner from the lateral supracondylar ridge into the centre of the defect (crossing the physis) using a posterior cruciate ligament targeting guide. 2 loops of 1 PDS suture were passed via these tunnels from the outside of the knee to the centre of the defect. Then a medial parapatellar approach to the knee joint was performed to formally expose the knee, two No. 2 Vicryl Bunnell sutures were passed across the femoral end of the ACL, and these sutures were placed into the PDS loops that had previously been passed into the knee. Pulling on the PDS sutures allowed the No. 2 Vicryl sutures to be passed through the tunnels previously drilled with the Beath pins and to be delivered to the lateral supracondylar ridge of the femur. The sutures were then tied to one another over a bone bridge thereby reducing the avulsed fragment back into its normal anatomical position (Figure 4). Postoperatively, the patient was splinted in extension for the first four weeks. At this stage, the patient was placed into a range of motion knee brace (allowing up to sixty degrees of flexion) and allowed to partially weight bear with crutches. At six weeks following surgery, the patient's brace was slackened to allow a full range of motion. Radiographs performed nine weeks following definitive fixation showed union of his fracture (Figure 5). He was allowed to ambulate free of his brace at this stage. At six-month follow up, the patient was able to ambulate without walking aids with a normal gait pattern and had a negative anterior drawer and Lachmann test and full restoration of knee flexion. No deformity or leg length discrepancy was noted at this follow up interval.

## 3. Discussion

Epidemiologically femoral end avulsions of the ACL may be subdivided into either purely cartilaginous avulsions [3–5] or osteochondral avulsion injuries [6–11], the latter being found in our patient whose case we have presented in this article. Furthermore, femoral end avulsion injuries may occur in isolation [3, 4, 7, 9–11] (as seen with the child we treated), simultaneously with a tibial end avulsion as well [5, 6], and also in tandem with a tibial end avulsion with a significant gap of time between the two injuries [8]. The youngest patient to have sustained such an injury was three years of age [3, 4]. The last two of these reports differed in their length of follow up with the latter reporting good outcome at 13-year follow up (as opposed to 10 weeks in the former report) with only slight laxity of the ACL. Almost universally these injuries occur as a result of relatively high-energy mechanisms as was the case with our patient.

Robinson and Driscoll [6] were the first group to report such an injury where it was seen in conjunction with a simultaneous avulsion of the tibial end of the ligament as well. The first report of an isolated avulsion of the femoral origin of the ACL was reported by Eady et al. [7]. Tohyama et al. [8] reported a case of consecutive avulsion fractures involving the ACL in which a tibial-sided avulsion fracture was successfully treated and was followed 35 months later by a second injury in which the femoral attachment of the ACL was avulsed.

Our technique of repair was in essence similar to that described by Robinson and Driscoll [6] with our transosseous tunnels for passage of the sutures attached to the femoral end of the ACL crossing the physis. As noted in the article by Corso and Whipple [3], concerns have been raised in treating such injuries using transosseous tunnels which cross the physis. In the report by Robinson and Driscoll [6] whose tunnel configuration we also utilised, the authors, however, reported no resulting distal femoral deformity. To counteract possible iatrogenic growth disturbance from transphyseal methods of ACL fixation, transepiphyseal methods of fixation have been advocated [12]. The technically demanding nature of this method of fixation mandates that surgery be performed by soft tissue knee surgeons with expertise in the treatment of ACL injuries in the skeletally immature.

In summary, the case presented in this paper illustrates the pitfalls that may be encountered when treating children presenting with acute injuries to the knee.
